# Probiotics and oral health: A systematic review

**DOI:** 10.4317/medoral.21494

**Published:** 2017-04-08

**Authors:** Maria Seminario-Amez, Jose López-López, Albert Estrugo-Devesa, Raul Ayuso-Montero, Enric Jané-Salas

**Affiliations:** 1 DDS. Student of Master in Oral Medicine, Surgery and Implantology. School of Dentistry. University of Barcelona, Spain; 2 PhD, DDS, MD. Professor of Oral Pathology. School of Dentistry, Barcelona University- Hospital Odontologíco Universidad de Barcelona. Oral Health and Masticatory System Group (Bellvitge Biomedical Research Institute) IDIBELL, University of Barcelona, L’Hospitalet de Llobregat, Barcelona, Spain; 3 PhD, DDS. Department of Odontoestomatology. School of Dentistry, University of Barcelona. University Campus of Bellvitge, Barcelona, Spain. / Oral Health and Masticatory System Group (Bellvitge Biomedical Research Institute) IDIBELL, Barcelona, Spain

## Abstract

**Background:**

Probiotics are microorganisms, mainly bacteria, which benefit the host’s health. Many studies support the role of probiotics as a contributor to gastrointestinal health, and nowadays many authors are trying to prove its influence in oral health maintenance.

**Objectives:**

To review the published literature with the purpose of knowing the importance of using probiotics as a preventive and therapeutic method for oral infectious diseases management.

**Material and Methods:**

An electronic search in PubMed database with the keywords “oral health AND probiotics AND dentistry” was conducted. The inclusion criteria were: randomized clinical trials (RCTs) that assess the action of any probiotic strain in the treatment and / or prevention of an infectious oral disease, RCTs that assess the action of any probiotic strain on counting colony forming units (CFU) of oral pathogens, systematic reviews and meta-analysis. The Jadad scale was used to assess the high quality of RCTs.

**Results:**

Fifteen articles were considered for this review. Of which, 12 were RCTs of good / high quality (Jadad scale), two meta-analysis and one systematic review.

**Conclusions:**

The literature reviewed suggests probiotics usage could be beneficial for the maintenance of oral health, due to its ability to decrease the colony forming units (CFU) counts of the oral pathogens. However, randomized clinical trials with long-term follow-up periods are needed to confirm their efficacy in reducing the prevalence/incidence of oral infectious diseases. Furthermore, the recognition of specific strains with probiotic activity for each infectious oral disease is required, in order to determine exact dose, treatment time and ideal vehicles.

** Key words:**Probiotics, periodontal diseases, dental caries, oral health.

## Introduction

Periodontal disease and dental caries are one of the most prevalent and important health problems worldwide (1,2). The treatment of these diseases or their complications may require systemic use of antimicrobial drugs; which trigger gastrointestinal side effects due to broad spectrum antibiotics (3), bacterial resistance and allergic reactions (4-6). For this reason, many authors have proposed alternative therapies that can offer satisfactory results without causing potential risks to the patient (7).

Probiotics are defined as nonpathogenic live microorganisms that, when administered in adequate amounts in foods or as dietary supplements, confer benefits to the host’s health (6,8-10). They were first proposed by Elie Metchnikoff, (Nobel Prize in Physiology and Medicine - 1908), who developed the theory that the Bulgarian population had greater longevity due to the consumption of fermented products containing lactic acid bacteria that improved gastrointestinal health (5,6,8). Since then, many publications refer to the use of probiotic strains (mainly *Lactobacilli* and *Bifidobacteria*) in the maintenance of gastrointestinal, genitourinary and oral health through providing the balance of these ecosystems (5,6,8-11).

The oral cavity is a microbiological medium that needs homeostasis (6,10). When factors such as time, poor oral hygiene, diet and immunodeficiency alter that balance, infectious diseases can develop and might require a complex management because of their polymicrobial nature (11,12). Microorganisms and toxins necessary for the destruction of oral tissues are organized in a thin film known as *biofilm* (6,13). This is deposited on the hard tissues of the oral cavity: enamel and cementum, and the dental implant surface (14). In microbiological terms, we can say that is the result of processes such as bacterial adhesion, aggregation and co-aggregation to colonize the oral cavity (14-16). The aim of this article is to review the published literature with the purpose of knowing the importance of using probiotics as a preventive and therapeutic method for oral infectious diseases management.

## Material and Methods

An electronic literature search in PubMed database between March and May 2016, with the keywords “probiotics AND oral health AND dentistry” was conducted. No time filter was used to limit the years of publication. The inclusion criteria were: randomized clinical trials (RCTs) that assess the action of any probiotic strain in the treatment and/or prevention of an infectious oral disease, RCTs that assess the action of any probiotic strain on counting colony forming units (CFU) of oral pathogens. Only those with a Jadad > 3 (17) were considered. Systematic reviews and Meta-analyzes were also included.

## Results

**Figure 1:**
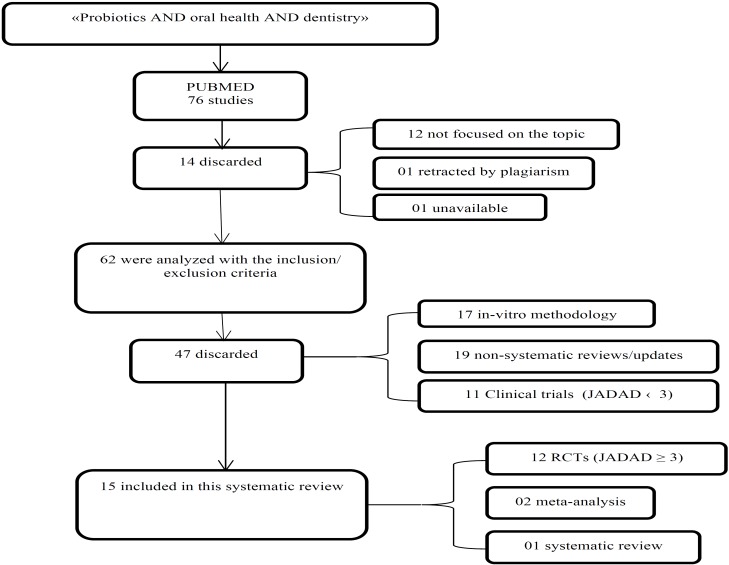
Selection process of the studies included in this systematic review.

An initial search resulted in seventy six items. After reading all abstracts, 12 studies were eliminated because they were not focused on the topic, 1 for being rejected by plagiarism and 1 for being unavailable. Seventeen *in-vitro* studies and 19 non-systematic reviews/updates were eliminated because they did not coincide with the inclusion criteria. In addition, 11 RCTs were discarded for not having a Jadad > 3 (Fig. 1). A total of 15 articles were considered for this review. Of which 12 were RCTs ( Table 1), 1 systematic review and 2 meta-analyses. RCTs included in this study (18-29) had a total of 1291 patients (n=1291). Of which, 380 patients were older than 18 years old and 911 were minors. Six studies used only *Lactobacilli* probiotic strains (19,20,23,27-29), 3 studies *Bifidobacteria* (24-26), 1 used *Streptococci* (18), and 2 studies used mixtures of probiotic strains (21,22). Seven RCTs evaluated the influence of bacteriotherapy in the colony forming units (CFU) counts of *S. Mutans* and dental caries (20-26), 4 in periodontal disease (18,19,28,29) and 1 in the CFU counts of *Candidas* (27). Four RCTs employed dairy products as a vehicle for the administration of probiotics (20,21,23,24), 6 used oral tablets (18,25-29) and 2 used powder sachets (19,22). Of the latter, Jindal G *et al.* (22) used it diluted as a mouthwash. Eight studies had an intervention time equal or greater than 8 weeks (18,19,20,23,25-27,29). The remaining four studies lasted 7 to 18 days (21,22,24,28). In 6 studies, patients were followed up only during the intervention time (23-25,27-29). Laleman I, *et al.* (18) and Ashwin D *et al.* (21) did a 6 months follow-up, Wattanarat O, *et al.* (20) and Morales A *et al.* (19) did a 1 year follow-up and Taipale T *et al.* (26) a 4 years follow-up.

**Table 1:**
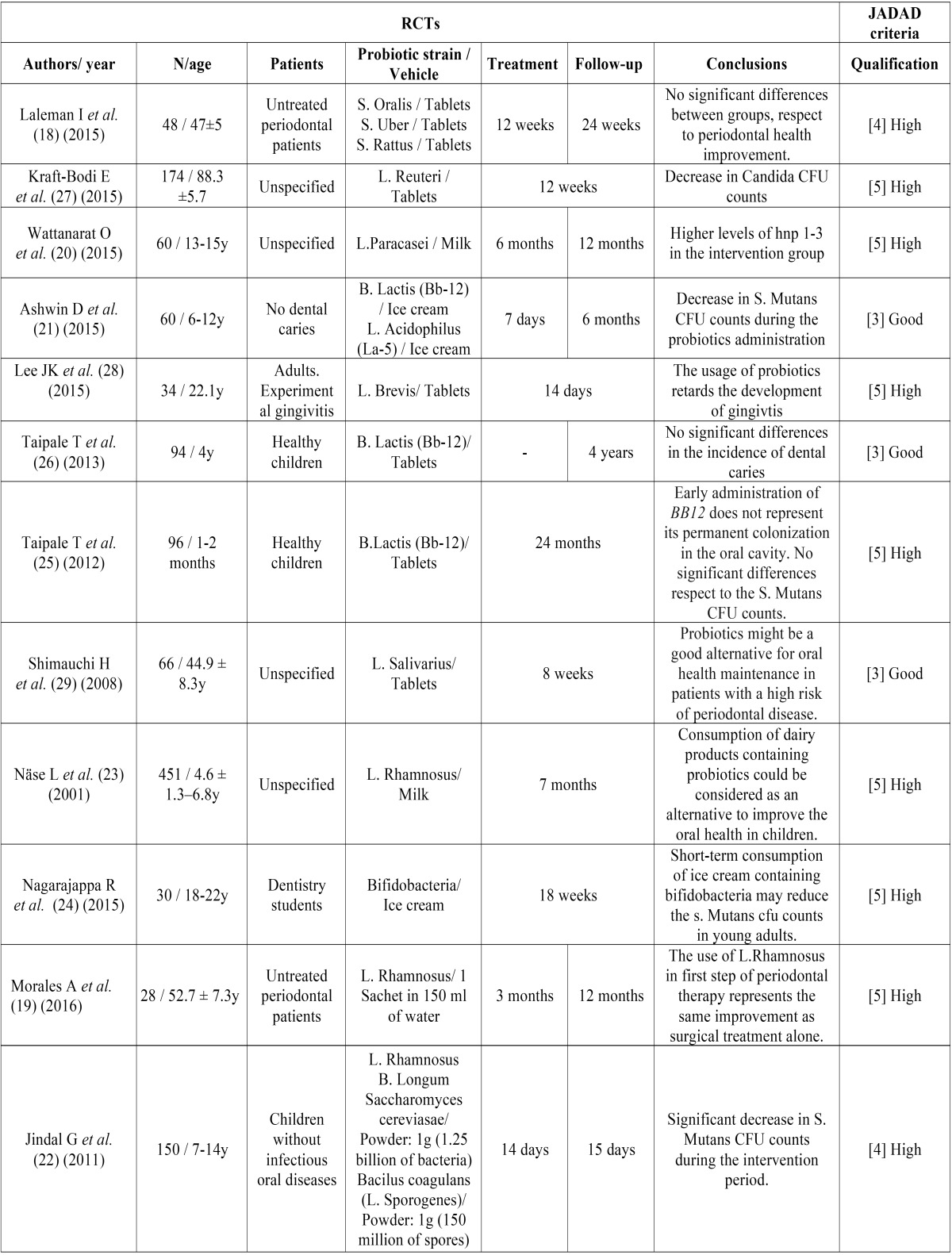
RCTs considered, due to their JADAD qualification.

## Discussion

The mechanism of probiotics in the oral cavity is not clearly established. They are associated with decreased CFU counts of cariogenic pathogens and the inhibition of periodontal pathogens (18,19,30,31). Furthermore, they modulate the inflammatory response (humoral and cellular) and produce substances such as lactic acid, hydrogen peroxide and bacteriocins (antimicrobial agents produced by lactic acid bacteria, whose action provides them of the probiotic effect) (8,13,30). Most of the studies reviewed mention its ability to compete with pathogens for adhesion surfaces and nutrients, causing the displacement of the latter ones (15,16).

None of the studies reviewed reported side effects associated with this bacteriotherapy (18-29). However, Taipale T, *et al.* (26) explained that in their study 2 patients from the intervention group with probiotics were removed because of gastrointestinal discomfort and 2 patients from the control groups with placebo were removed for the same reason and atopic eczema. None of the authors used probiotics as a therapeutic, preventive or adjuvant method in immunosuppressed patients. Probably because of ethical reasons associated to the inherent risks of their systemic condition. Rao Y, *et al.* (32) and Saraf K, *et al.* (33) referred to the use of this bacteriotherapy in patients with HIV and cancer to boost the immune system and fight allergies; however, they recognized that their use in oral health maintenance has not yet been tested.

The two most important topics reviewed in the studies included in this systematic review are periodontal disease and dental caries, which are described separately.

* i.- Probiotics and Dental caries

Dental caries is a multifactorial disease. Its pathogenesis involves the microbiota (mainly, *S. Mutans*), the host and its immune mechanisms, as well as the diet among other factors (1,6,11). Even though all these factors have to intervene in order for dental caries to develop, microbiological factors are still the leading cause of this disease. Therefore, it is thought that this bacteriotherapy could influence positively in the prevention of this disease (6,11,31,34). In the meta- analysis performed by Laleman I, *et al.* (11), of the 725 studies found in the databases PUBMED and ISI Web of KnowledgeSM, only 19 met the inclusion criteria and were used for the descriptive analysis. Of those, only 2 assessed the caries prevalence after probiotics therapy. Given this limitation, they had to focus on the CFU counts of *S. Mutans* and *Lactobacilli* before and after the probiotics treatment. This meta- analysis shows that when comparing the intervention group with the control group before and after the bacteriotherapy, there is a significant decrease in CFU counts of *S. Mutans*, which does not happen with the CFU counts of *Lactobacilli*. In addition, after treatment with probiotics, the intervention group had a greater number of patients with low levels of *S. Mutans* CFU counts (< 105 CFU / ml) and fewer patients with high levels (> 106 CFU / ml), which does not occur in the control group, neither when the same comparison with *Lactobacilli* CFU counts was performed. Of the 19 studies used for the descriptive analysis of this meta-analysis; only two had low risk of bias; ten had a moderate risk, and seven high risk [based on assessment criteria from the Cochrane Center, Consort Guide (35), Delphi list (36), and the list proposed by Van der Weijden, *et al.* (37)]. The high probability of bias and the number of studies used, make us think that the results of this study may not be applicable. However, the authors explained that this limitation is based on the fact that the studies published have very heterogeneous methodologies; due to the study design, treatment and follow-up time, probiotic strains, concentrations and vehicles used, as well as the population selected for the study, ages and local contributing factors (orthodontic appliances and dental prostheses). Laleman I, *et al.* (11); Wattanarat O, *et al.* (20); Jindal G, *et al.* (22); Nase L, *et al.* (23); Nagarajappa R, *et al.* (24) concluded that the use of probiotics can reduce *S. Mutans* CFU counts during the time they are used, and this could have a preventive effect on the development of dental caries; however, the lack of long-term follow-up periods makes it impossible to suggest that the effect continues after stopping the bacteriotherapy, or its influence on the prevalence/incidence of dental caries. In addition, it has to be determined which would be the appropriate strain, time of treatment, concentration and the ideal vehicle to be used. On the other hand, Taipale T, *et al.* (25) conducted a 24 months double double-blind study in a population of 96 one month old children, which were divided into three randomized groups, receiving probiotics (*B. Lactis BB -12*), xylitol or sorbitol. They concluded that early administration of this probiotic strain did not represent its permanent colonization in the oral cavity and that the CFU counts of *S. Mutans* were not significantly affected. The same group published a post-clinical trial of these patients at 4 years-old; confirming that in that population probiotics therapy did not exercise significant differences in the incidence of dental caries, compared to the xylitol and sorbitol control groups. Furthermore, in the systematic review made by Twetman *et al.* (8) which reviewed three RCTs that used *L. Rhamnosus* and milk as a vehicle for the prevention/control of dental caries, they concluded that despite the encouraging results about the preventive capacity of probiotics in dental caries, it would be premature to give any clinical recommendation due to the lack of RCTs with a long-term follow-up period. Gruner D, *et al.* (31) conducted a meta-analysis of 50 clinical trials published between 2001 and 2015, with a total of 3247 patients (n=3247). The authors explained that probiotic therapy significantly reduces the *S. Mutans* CFU counts (<104 UFC/ml), and that *Bifidobacteria* are the most significant contributors to this effect. However, if the limit is 105CFU/ml or 106UFC/ml, there are no significant differences with the control group. Unfortunately, according to the authors, studies that support those data were not clear or had a high risk of bias. For that reason, they considered the existing clinical evidence inconsistent to make recommendations about the use of probiotics in the treatment or prevention of dental caries. Nevertheless, as they did not found association between this bacteriotherapy and the occurrence of adverse effects, they did not recommend its contraindication. The same authors analyzed that RCTs which evaluated *Lactobacilli* (cariogenic bacteria) colonization, showed an increase in CFU counts; probably because of the use of probiotic strains of the same genus. Therefore, they proposed that future RCTs should evaluate cariogenic *Lactobacilli* counts and probiotic *Lactobacilli* counts separately. Of the 50 clinical trials included in this meta-analysis, 45 used Lactobacilli and 12 used *Bifidobacteria*; which coincides with our review.

* ii.- Probiotics and Periodontal Disease

Imbalance between the saprophytic and pathogenic flora of the oral cavity, in a susceptible host, can result in periodontal disease (2). Debridement treatment of this disease can be surgical or nonsurgical and in some cases systemic administration of antimicrobials is required (19). Due to bacterial resistance associated with the prescription of these drugs, new alternatives for periodontal health maintenance are needed (6,19). The mechanism of probiotics is associated with the production of substances (lactic acid, hydrogen peroxide and bacteriocins) and the modification of the *biofilm* (38,39). It has also been mentioned a possible decrease of pro- inflammatory cytokines, collagenases, elastases and prostaglandins E2 levels (40). Piwat S, *et al.* (15) conducted an *in-vitro* study that, despite not meeting our inclusion criteria, deserves to be commented because it is one of the few jobs that explains the adhesion, aggregation and co-aggregation mechanisms of probiotic bacteria, and their influence in the probiotic effect. In theory, probiotic bacteria attach to the oral tissues more strongly than pathogens, being able to compete for adhesion surfaces. From this process, bacterial aggregation and co-aggregation are triggered, thus producing a new “*biofilm*” (15,19,30,39). Morales A, *et al.* (19) conducted a clinical study to evaluate the use of probiotics as an adjuvant therapy in nonsurgical periodontal treatment in patients with previously untreated chronic periodontitis. Although the intervention group (14 patients) had lower probing depth than the control group, even at one year follow-up, this difference was not statistically significant. Therefore, they concluded that the adjunctive use of *L. Rhamnosus*sachets provides same clinical results as scaling and root planning alone. Shimauchi H, *et al.* (29) found that probiotic treatment with *L. Salivarius* improves plaque index and probing depth in smokers. For that reason, they concluded that this bacteriotherapy is a good alternative for oral health maintenance in patients with high risk of periodontal disease. Laleman I, *et al.* (18) conducted a clinical trial in which 48 adults with untreated periodontal disease were divided into two groups. The intervention group received *S. Oralis*, *S. Uberis* y *S. Rattus*, and the control group a placebo. Both groups underwent scaling and root planning and had to take 1 tablet (depending on the group they belonged) twice a day during 12 weeks as an adjunctive therapy. At the end of follow up period (24 weeks), they found a significant improvement (*p* < 0.05) in periodontal health of both groups. However, there were no significant differences between groups at 12 weeks of treatment, neither at 24 weeks of follow up. Despite they also found a decreased in *Prevotella Intemedia* CFU counts at 12 weeks of treatment in the intervention group compared to the baseline counts (*p*= 0.02), they concluded that there were no significant differences between groups and that the usage of those probiotic strains as an adjuvant therapy after scaling and root planning has no clinical nor microbiological relevance. In another study, Laleman I, *et al.* (6) highlighted that the usage of probiotics does not replace the daily oral hygiene technique. In the meta-analysis of Gruner D, *et al.* (31), that we have already mentioned, they included 3 clinical trials which evaluated the influence of probiotic therapy with *Lactobacilli* in periodontal pathogens counts (*Agregatibacter Actinomycetemcomitans, Porphyromana Gingivalis and Prevotella Intermedia*) and no statistically significant differences were found compared with the control group. Nevertheless, when clinical signs such as gingival index and bleeding on probing are assessed, they found an improvement in the interventions groups, which did not occur with plaque index. This is the reason why they refered that the probiotic effect may lie in the host response, rather than in the periodontal pathogens. However, the risk of bias of the considered clinical trials was high.

## Conclusions

Probiotics are a kind of bacteriotherapy which, according to the literature reviewed, provides a decrease in CFU counts of cariogenic pathogens (*S. Mutans*). Regarding periodontal disease, the studies included in this review reported a clinical improvement of bleeding on probing, probing depth and gingival index, but no significant difference in CFU counts of periodontal pathogens. Anyway, it is important to highlight that these diseases have a multifactorial etiology, which means that reducing the CFU counts does not ensure their absolute control. RCTs with homogeneous methodologies and long-term follow-up periods are needed to confirm their contribution in the management of these diseases and their influence in their prevalence. Furthermore, the recognition of specific strains with probiotic activity for each infectious oral disease is required in order to determine exact dose, treatment time and ideal vehicles.
